# Drug Interactions for Inhibition or Killing of *Coccidioides* Species

**DOI:** 10.3390/jof12030181

**Published:** 2026-03-03

**Authors:** David A. Stevens

**Affiliations:** 1 Division of Infectious Diseases and Geographic Medicine, Stanford University Medical School, Stanford, CA 95304, USA; stevens@stanford.edu; Tel.: +1-408-867-5968; 2 Foundation for Research in Infectious Diseases, P.O. Box 2734, Saratoga, CA 95070, USA

**Keywords:** *Coccidioides*, drug interactions, drug susceptibility, minimum fungicidal concentration, synergy, antagonism

## Abstract

Coccidioidomycosis is of concern because of its rising incidence, the spread of its endemicity, the virulence of its progressive infections, and the difficulty of achieving successful therapy, particularly durable responses. As a result, clinicians will often attempt combination therapy with amphotericin B and an azole. However, the effects of such combinations on the inhibition or killing of the fungus are largely unknown. The present report details studies of these drug interactions against *Coccidioides* in vitro with checkerboard arrangements, with 239 interactions for inhibition and 76 for killing detailed. The frequency of synergy in inhibition was similar for the five most common azoles, accounting for about one third of outcomes. Synergistic and/or additive effects were noted about half of the time. Of concern, antagonism in inhibition was noted in 14–23% of cases (slightly less in the interactions for killing). These data inform clinicians of the probability of the type of interaction that may be seen with a particular azole, but of more interest in therapy selection are the interactions with the patient’s individual isolate. It is emphasized that the interactions detailed are described for in vitro studies, and must be substantiated by studies in vivo; however, the data highlights the framework needed for validation studies, and highlights the importance of an awareness of the potential downsides of such combinations in individual patients.

## 1. Introduction

Coccidioidomycosis is of increasing interest because of its rising incidence [1], and also because of its spreading geographic distribution [2]. Progressive coccidioidomycosis has been regarded as among the most difficult to treat, even in non-immunocompromised hosts. In immunocompromised hosts, such infection is frequently fatal, even with antifungal treatment [3]. Evidence for the difficulty of treatment includes the extraordinary length of therapy compared to other mycoses, recommended by experts for the progressive forms of the disease [4]. Related to that, in the randomized trial of coccidioidal therapy (in noncompromised hosts) [5], a clearcut difference was only evident when observation was extended to 12 mos. of therapy, as opposed to the initial intent to analyze at 9 mos. For meningeal coccidioidomycosis, the standard of care is lifelong azole suppressive therapy, if suppression is successful [6,7].

The difficulty of treatment may be explained by the inherent virulence of this fungus, and/or because the in vivo form of this dimorphic fungus is spherules, which are large forms that are difficult for phagocytes to ingest and also have antiphagocytic external defenses [8]. Coccidioidal parenchymal lesions are characteristically suppurative and show granuloma formation, both of which retard antifungal drug penetration [9].

Amphotericin B was the first somewhat effective anti-coccidioidal therapy, is rapidly acting, but its toxicity [10] and requirement for parenteral therapy are obstacles to its use. If amphotericin is selected for initial therapy, clinicians are desirous to achieve the benefits of combining this treatment with markedly less toxic azole drugs, transitioning to oral azole monotherapy for the long treatment durations required. Dissatisfaction with the results of azole monotherapy [7,11] have also led clinicians to try combination therapy. These types of clinical approaches are evidenced in the frequency of such test requests in the results summarized in this study. In some published series discussing the manifestations of coccidioidal disease, all the patients described received a combination of amphotericin and an azole [12,13]. Despite a general enthusiasm for combination antifungal therapy [14], there is no literature on the effect on inhibition or killing of *Coccidioides* of combination therapy. I present here my laboratory’s experience with in vitro studies of drug combinations against *Coccidioides*, including 239 interactions for inhibition and 76 for killing.

## 2. Methods

### 2.1. Isolates and Testing Plan

The isolates studied were 67 *Coccidioides* spp. isolates, as identified by the laboratory sending them, in mycelial form, to my research laboratory at the California Institute for Medical Research, San Jose, CA, USA, from 1991 to 2023. This laboratory maintains a State and Federal clinical laboratory license and has Biohazard level 3 certification (required to handle coccidioidal isolates). These isolates were sent by clinicians, >90% from California, requesting susceptibility results. However, it is almost always impossible, except in instances of focal epidemiological outbreaks, to know at what location the patient acquired their coccidioidal infection. The drugs tested were at the request of the sending physician, hospital, or clinic. With the single exception of a combination study with sertraline, the drug interaction studies requested were amphotericin with azoles, the logic being that azoles, having the same mechanism of action, would be unlikely to have synergistic or antagonistic interactions with each other (although possibly additive or indifferent interactions could be hypothesized). A logical reason for the information requested would be a desire to concurrently start an azole with amphotericin, hoping for a benefit from the more fungicidal, faster-acting, and time-tested drug amphotericin, and at least some benefit from the azole; provided an experience with the azole that was useful at that time, amphotericin, the more toxic [10] drug (requiring parenteral therapy), could be discontinued in favor of monotherapy with an oral azole.

Limited clinical information about the patients was available, but overwhelmingly, the requests appeared to have originated prior to the start of any prolonged therapy course. The patients’ subsequent course was in most instances unknown to us. The studies tabulated here were on one isolate/patient, although in a single instance, a second isolate was submitted from a patient later in the patient’s clinical course; in that instance, the 2 sets of studies had identical results. Minimal inhibitory concentrations (MICs) were determined on all isolates tested. The MIC data on these isolates is part of an extensive manuscript in preparation, comparing our laboratory results with those of the Univ. of Texas, San Antonio laboratory (only MICs were tested), and also examining temporal MIC trends in both laboratories [15].

### 2.2. Testing Methodology

Broth macrodilution was performed in tubes as previously described [16,17,18,19]. In brief, arthroconidia were harvested from mycelia and suspended in broth. A novel feature, as viewed presently, was the medium used throughout for the studies reported, SAAMF [20], chosen because it is fully defined (and lacking any components that could antagonize drugs, particularly new ones with as yet undefined mechanisms of action) and because we have found that all *Coccidioides* isolates grow well in it. RPMI1640 is another fully defined medium [16], originally introduced to facilitate the culture of human cells [21], and which also promotes the growth of most fungi. We performed a pilot study of MICs on 3 isolates that showed that, changing nothing else in the testing procedure as detailed, but substituting RPMI1640 for SAAMF, the results were similar; we elected to continue using SAAMF for coccidioidal studies, in part to have a uniform data set over time. Pure drug powders were obtained from the manufacturers. Testing of amphotericin B is performed with the deoxycholate form everywhere, as amphotericin B is the active component in lipid-formulated amphotericin B preparations as well [16]. The range of drug concentrations to be tested was initially set to encompass blood concentrations derived from these drugs’ known pharmacokinetics in humans; these ranges are shown in Supplementary Table S1. If pharmacokinetics were unknown at the initiation of testing a drug, then a broader range of drug concentrations was instituted. The range was extended further if occasional isolates were apparently resistant to amphotericin or the azole in vitro, to attempt to capture an MIC value in both outer rows of the checkerboard (arrangement to be described below).

To evaluate the combination activity of antifungal drugs, a traditional checkerboard assay [22,23] was performed, and interpreted as previously described [24,25,26,27,28,29,30,31], determining the fractional inhibitory concentration index (FICi). In brief, the drugs tested were diluted twofold over the range tested. A row of MIC tests of tube dilutions of each drug alone form the two outer rows of the checkerboard array. Each tube within the matrix of the checkerboard contains the same final concentration of both drugs as the two corresponding “MIC, drug alone” outside rows that “intersect” in the matrix at that tube. All tubes have a final volume of 2 mL, to which was added 0.1 mL at 10^3^ arthroconidia/mL. Before incubation, all tubes are clear. The endpoints comprise tubes that are clear at the end of incubation. A drug-free tube (inoculum growth control), an inoculum-free tube (reagent sterility control), and tubes containing the drugs to be tested but with a known pansensitive *Candida kefyr* (recently reassigned taxonomically as *Kluyveromyces marxianus*) isolate (drug potency control) were concurrently established. Reproducibility of MIC and MFC results were established annually with a sample of isolates, per California State Dept. of Health and Federal standards and inspections of our laboratory.

The tubes were incubated at approximately human body temperature (35 °C to 37 °C) for 1 wk. (at this time, control in the drug-free tube was deemed 4+/4, semiquantitatively), and the MIC endpoint was determined by the clear tube at the lowest drug concentration in the single drug (outside) rows. The Fractional Inhibitory Concentration (FIC) for a drug in combination tubes is the result of dividing the MIC of the drug alone against a microbe into that drug’s concentration in a combination tube showing inhibition. The FICi is determined by the sum of the FICs for both drugs, in the combination tube with the lowest sum of FICs [32,33,34]. Interaction effects were categorized as synergism (FICi < 1), additive (1 < FICi < 2), indifference (FICi = 2), or antagonism (FICi > 2) [24,27,28,29,30,31]. In words, according to this definition, synergism occurs when a combination tube produces the desired result at concentrations lower than both drugs’ MICs alone. An additive effect is when there is enhancement of ≥1 drug in the interaction but this does not meet the criteria of synergy; an example of this is when the tube defining the FICi comprises the MIC of drug A and one quarter of the MIC of drug B—in this example, the action of drug B is enhanced by drug A, but not vice versa. Indifference indicates no contribution of either drug’s activity to that of the other (each drug in combination acts as if the other is absent); antagonism indicates that more than both drugs’ MICs in combination are required to produce the desired effect, with their combined effects less than the effect of either drug alone. Classifications of FICi have been somewhat variable in the literature. A common issue is the classification of synergy, with some authors’ recommending an FICi < 0.5 to demarcate synergy, the major reason being that an FICi 0.5 to <1 could theoretically result from a small dilution error. We have chosen, according to our previous efforts [24,27,28,29,30,31], to follow here the strict arithmetic system present in the literature [25,26] of using an FICi < 1 to indicate synergy. However, for convenience, in our Results, I have also combined the categories of synergy and additive, the two “positive” categories (FICi < 2), together, for concurrent inspection. Some other possible combinations of our categories can easily be similarly calculated by the reader from the tables in this paper.

### 2.3. Killing

In many instances, drug interactions for killing were also requested. After vortex-mixing at the end of incubation, fifty microliters were removed from the clear tubes and cultured on SAAMF agar for 1 wk. at that temperature, and the agar was enumerated for coccidioidal colonies. If there were no colonies, this implied ≥99% killing in that tube. The Fractional Fungicidal Concentrations and Fractional Fungicidal Index (FFCi) were calculated and interpreted in the same way as described above for FICi. The MFCs of individual drugs are presented in a separate publication [35].

## 3. Results

### 3.1. Drug Interactions for Inhibition

The overall frequency of interactions for synergy were strikingly similar for the azoles (35–41%), although that for posaconazole appeared to be slightly less. A factor for posaconazole is that it appears to be a more potent inhibitor alone of *Coccidioides*, compared to other azoles, demonstrable in vitro and in animal models [35,36], in patients [37], and even in clinical salvage therapy of failures from prior other therapies [38]; this level of activity could make it more difficult to show a synergistic interaction with amphotericin B. A possible clue to answer this hypothesis could be the higher percent of additive and indifferent interactions for posaconazole than the other azoles, possibly reflecting shifts from the synergy category. The overall interactions of antagonism were also generally similar for all azoles (14–18%), although this outcome was slightly more common with fluconazole (23%). If synergy and additive outcomes are regarded as either positive to a lesser or greater extent, or if these two outcomes are regarded as a continuum that might be difficult to separate using the checkerboard technique, the last row of the table (combining these two outcomes) reveals a narrow range for all five azoles of 42 to 54%. Not shown is one interaction study requested for amphotericin B–sertraline, which showed synergy in inhibition.

With respect to patterns for individual isolates, none could be seen among any of the studies. That is, analyses of interaction studies on individual isolates, when >1 interaction study on the same isolate was requested, revealed no instance where antagonism was seen for all the different drug interactions ordered, and rarely was it the case that all interactions ordered on that isolate showed synergy. Overwhelmingly, the pattern was some mix among the four types of interaction for the different drugs for individual isolates.

### 3.2. Drug Interactions for Killing

The frequency of antagonism for killing was similar (11 to 19%) for fluconazole, voriconazole, and posaconazole, but lower for isavuconazole and itraconazole. The frequency of antagonism for killing was lower for every combination compared to antagonism for inhibition (Table 1), except posaconazole, which was essentially the same.

The frequency of synergy for killing was similar for all azole combinations (29 to 36%), except posaconazole, which was lower. The frequency of synergy + additive results was similar for all azole combinations (38 to 55%). Again, there may have been a shift from synergy into additive or indifferent results for posaconazole apparent, a possible explanation of which has already been discussed regarding inhibition. The similarity of posaconazole in terms of the synergistic + additive results for killing to those of the other azoles, despite the lowest percent for synergy in killing, supports the previous possible explanation of posaconazole’s shifts in category.

The frequency of synergy for killing was similar for all azoles compared to synergy for inhibition (Table 1), except for posaconazole, which was lower. The synergistic + additive results for killing (Table 2) were similar to the synergistic + additive results for inhibition (Table 1) for voriconazole, posaconazole, and isavuconazole, but lower for fluconazole and itraconazole.

## 4. Discussion

In this report, it is shown that five common azoles are roughly similar in their interaction with amphotericin against *Coccidioides*, with roughly one third showing a synergistic interaction for inhibition or killing and almost half showing at least some potentiation (synergy and/or additive effect), as defined herein. However, a small but substantial fraction remains in which the drugs antagonize each other. The extensiveness of this data provides an overview which informs clinicians of the probabilities of what the interaction might be when an azole is offered in conjunction with amphotericin. However, what a clinician most wants to know is what the likely interactions are for their patient’s own isolate, and this is the information that was provided to the requestors. The potential limitations of this information are discussed below, but an overall viewpoint is expressed in a classic article [22]:

*Although very few … diagnostic laboratories are currently testing the activity of antibiotics in combination, such practice is going on daily on the wards of virtually every hospital where patients are receiving two … antibiotics simultaneously; the test is inside the patient. The possibility that the laboratory can be of service in determining the most advantageous combinations or providing evidence that there may be antagonism could certainly be very useful in the practical management of … antibiotics for patients with infectious diseases.*

Firstly, it must be recognized that these are in vitro interactions, whereas, in vivo, the pharmacology of the drugs becomes another essential consideration. What are the relative concentrations of the drugs in the patient, particularly in the infected tissues? What are the pharmacologic drug–drug interactions that might affect each drug’s pharmacology, and/or the side effects experienced? The definitive resolution of the effects of combination therapies vs. monotherapy must occur in trials in vivo, in animal models, and in randomized clinical studies. The present data provide an opening to inform this continuum. Fortunately, there are time-tested animal models [39,40] that could give important insights, although differences between animal and human dosing and pharmacology must be taken into account. The utility of the present data could be briefly summarized by assuming that if two drugs act synergistically in vitro, it is unlikely that this would be detrimental to efficacy in vivo, and quite possibly it would be beneficial; however, if two drugs antagonize each other in vitro, it is unlikely that this would be beneficial to efficacy in vivo, and likely it would be detrimental in vivo.

*Other limitations of the present study*: There are two species of *Coccidiodies*, *immitis* and *posadasii*, and as our isolates are overwhelmingly from California, *C. immitis* likely is predominant [41]. However, no differences in drug susceptibilities between the two species have been found [42]. The present study was performed on mycelial *Coccidioides*, not the parasitic phase. This is because only arthroconidia of the mycelial phase can logistically be tested (because of difficulties in growing spherules and endospores in vitro, and even greater difficulties in quantitating an inoculum of these elements). This limitation is true of all the prior coccidioidal drug testing in vitro reported in the literature. It is unclear whether there are differences in phase susceptibilities for an individual isolate. In addition, differences in methodology used by various laboratories could impact the results obtained. It would also be important to identify the genomic determinants of the variations in the interactions detailed here.

## Figures and Tables

**Table 1:** Drug interactions for inhibition (number of isolates, % of interactions for a drug).

Amphotericin B with	Fluconazole	Itraconazole	Voriconazole	Posaconazole	Isavuconazole
Synergy	25 (**40%**)	18 (**36%**)	17 (**35%**)	10 (**21%**)	12 (**41%**)
Additive	8 (**13%**)	9 (**18%**)	4 (**8%**)	10 (**21%**)	3 (**10%**)
Indifferent	15 (**24%**)	16 (**32%**)	19 (**39%**)	21 (**44%**)	10 (**35%**)
Antagonism	14 (**23%**)	7 (**14%**)	9 (**18%**)	7 (**15%**)	4 (**14%**)
TOTAL	62	50	49	48	29
Synergy + additive	33 (**53%**)	27 (**54%)**	21 (**43%**)	20 (**42%**)	15 (**52%**)

**Table 2:** Drug interactions for killing (number of isolates, % of interactions for a drug).

Amphotericin B with	Fluconazole	Itraconazole	Voriconazole	Posaconazole	Isavuconazole
Synergy	6 (**33%**)	4 (**29%**)	5 (**29%**)	2 (**13%**)	4 (**36%**)
Additive	1 (**6%**)	1 (**7%**)	4 (**24%**)	5 (**31%**)	2 (**18%**)
Indifferent	9 (**50%**)	8 (**57%**)	6 (**35%**)	6 (**38%**)	5 (**45%**)
Antagonism	2 (**11%**)	1 (**7%**)	2 (**12%**)	3 (**19%**)	0
TOTAL	18	14	17	16	11
Synergy + additive	7 (**39%**)	5 (**38%)**	9 (**53%**)	7 (**44%**)	6 (**55%**)

## Data Availability

Individual data is unavailable owing to privacy restrictions.

## Supplementary Materials

The following supporting information can be downloaded at https://www.mdpi.com/article/10.3390/jof12030181/s1: Table S1: Cmax from literature reviews.
