# Supplementary material for: Drug Interactions for Inhibition or Killing of Coccidioides Species
Source: J Fungi (Basel). 2026 Mar 3;12(3):181. doi: 10.3390/jof12030181 (PMC13027441; doi:10.3390/jof12030181)
Supplement: Supplementary file 1 [file jof-12-00181-s001.zip › jof-4142485 - Supplementary.pdf]

Table S1.Cmax's from literature reviews

From ref. 35.

DRUG	Cmax (mcg/mL)
Amphotericin B deoxycholate	0.5-5.4
Liposomal amphotericin B	14-47
Amphotericin B lipid complex	1.6
Fluconazole*	4.1-20
Itraconazole	0.3-1.3
Posaconazole	0.6-4
Isavuconazole	0.5-2.6
Voriconazole	2-4.6
Caspofungin	7-10
Anidulafungin	7
Micafungin	3.4-18

* with 400 mg oral dose
